# Worthwhile or Not? The Pain–Gain Ratio of Screening Routine cMRIs in a Maximum Care University Hospital for Incidental Intracranial Aneurysms Using Artificial Intelligence

**DOI:** 10.3390/jcm14124121

**Published:** 2025-06-11

**Authors:** Franziska Mueller, Christina Carina Schmidt, Robert Stahl, Robert Forbrig, Thomas David Fischer, Christian Brem, Klaus Seelos, Hakan Isik, Jan Rudolph, Boj Friedrich Hoppe, Wolfgang G. Kunz, Niklas Thon, Jens Ricke, Michael Ingrisch, Sophia Stoecklein, Thomas Liebig, Johannes Rueckel

**Affiliations:** 1 Department of Radiology, University Hospital, LMU Munich, 81377 Munich, Germany; 2 Institute of Neuroradiology, University Hospital, LMU Munich, 81377 Munich, Germany; 3 Department of Neurosurgery, University Hospital, LMU Munich, 81377 Munich, Germany

**Keywords:** artificial intelligence, intracranial aneurysms, screening

## Abstract

**Background:** Aneurysm-related subarachnoid hemorrhage is a life-threatening form of stroke. While medical image acquisition for aneurysm screening is limited to high-risk patients, advances in artificial intelligence (AI)-based image analysis suggest that AI-driven routine screening of imaging studies acquired for other clinical reasons could be valuable. **Methods**: A representative cohort of 1761 routine cranial magnetic resonance imaging scans [cMRIs] (with time-of-flight angiographies) from patients without previously known intracranial aneurysms was established by combining 854 general radiology 1.5T and 907 neuroradiology 3.0T cMRIs. TOF-MRAs were analyzed with a commercial AI algorithm for aneurysm detection. Neuroradiology consultants re-assessed cMRIs with AI results, providing Likert-based confidence scores (0–3) and work-up recommendations for suspicious findings. Original cMRI reports from more than 90 radiologists and neuroradiologists were reviewed, and patients with new findings were contacted for consultations including follow-up imaging (cMRI / catheter angiography [DSA]). Statistical analysis was conducted based on descriptive statistics, common diagnostic metrics, and the number needed to screen (NNS), defined as the number of cMRIs that must be analyzed with AI to achieve specific clinical endpoints. **Results**: Initial cMRI reporting by radiologists/neuroradiologists demonstrated a high risk of incidental aneurysm non-reporting (94.4% / 86.4%). A finding-based analysis revealed high AI algorithm sensitivities (100% [3T] / 94.1% [1.5T] for certain aneurysms of any size, well above 90% for any suspicious findings > 2 mm), associated with AI alerts triggered in 22% of cMRIs with PPVs of 7.5–25.2% (depending on the inclusion of inconclusive findings). The NNS to prompt further imaging work-/follow-up was 22, while the NNS to detect an aneurysm with a possible therapeutic impact was 221. Reference readings and patient consultations suggest that routine AI-driven cMRI screening would lead to additional imaging for 4–5% of patients, with 0.45% to 0.74% found to have previously undetected aneurysms with possibly therapeutic implications. **Conclusions:** AI-based second-reader screening substantially reduces incidental aneurysm non-reporting but may disproportionally increase follow-/work-up imaging demands also for minor or inconclusive findings with associated patient concern. Future research should focus on (subgroup-specific) AI optimization and cost-effectiveness analyses.

## 1. Introduction

Artificial intelligence (AI)-based diagnostic algorithms have been demonstrated to mimic/overcome the performance of healthcare specialists in a variety of different medical image analysis scenarios [1,2,3,4,5,6]. Nevertheless, highly sensitive algorithms, while effective in minimizing the rate of missed findings, also pose a risk of overdiagnosis and inappropriate follow-up diagnostics or treatments [2].

An AI-based screening of cranial magnetic resonance imaging (cMRI) scans for incidental intracranial aneurysms might be worthwhile for the following reasons. There is a relevant prevalence estimated to range from approx. 2% up to 7% [7,8,9]. Furthermore, most aneurysms remain asymptomatic; however, their potential rupture is a devastating subset of stroke; aneurysm-related subarachnoid hemorrhage (SAH) has mortality rates of 25–50% [10,11], as well as high morbidity implications in case of survival, with up to 50% of SAH survivors suffering from long-term disability [12]. An early diagnosis, facilitated by the growing number of cMRI examinations for various indications, allows for individualized patient counseling, which needs to balance the risks of aneurysm rupture and preventive treatment strategies (surgical clipping or progressively improving endovascular techniques) [13,14,15,16]. Medical imaging specifically acquired for aneurysm screening is not widely established and is typically reserved for a minority of high-risk patients [17]. However, advances in AI-based image analysis raise the question of whether implementing AI-driven screening on imaging studies obtained for other clinical indications could be valuable, particularly given prior study findings highlighting the substantial risk of incidental intracranial aneurysm non-reporting, even by experienced (neuro-)radiologists [18].

AI algorithms for the detection of intracranial aneurysms in time-of-flight magnetic resonance angiography (TOF-MRA) [19,20,21,22,23,24,25] or computed tomography angiography (CTA) [26,27,28] have been proposed by the scientific community. Nevertheless, many studies are limited to algorithm performance evaluation and suffer from methodological limitations, e.g., small sample sizes [19,23], reference standards of limited quality [19,22], and biases resulting from pathology-enriched validation cohorts that partially underrepresent small aneurysm sizes [23] and fail to reflect the real-world prevalence seen in routine clinical practice [20,21,24,25]. None of these studies specifically addressed the question of whether the implementation of an AI-powered routine screening of clinically acquired cMRI scans might be worthwhile in a real-world scenario.

In a previous pilot study involving a neuroradiologists’ 3T cMRI cohort of consecutive psychiatric patients, a commercial AI algorithm for TOF-MRA aneurysm detection has been validated, demonstrating promising results while also highlighting a substantial risk of incidental aneurysm underreporting [18]. The study presented here now additionally enrolled a representative 1.5T cMRI inpatient cohort of the general radiology department of the same maximum care university hospital; both cohorts were finally merged into a dataset of 1761 cMRI scans. Based on the TOF-MRA analysis using a commercially available AI algorithm, a cMRI re-assessment by consultant-level neurointerventionalists (reference standard), initial cMRI report reviews, and a prospective study arm including follow-/work-ups for suspicious findings by cMRI or catheter angiography as jointly discussed with the patients, this methodology allows balancing the benefits of an AI second-reader routine screening in terms of additionally uncovering clinically relevant findings with the resulting follow-/work-up imaging burden also caused by small or inconclusive findings. This analysis was applied to a study cohort that mirrors the routine clinical practice of a maximum care university hospital, including both general radiology and neuroradiology departments and aimed to quantitatively simulate the additional impact of an AI-assisted routine screening in comparison with initial cMRI reports of more than 90 different (neuro-)radiologists.

## 2. Materials and Methods

Ethical approval was obtained by the institutional ethics committee according to the Declaration of Helsinki (approval number 23-0342). Patients’ informed consent for retrospective cMRI inclusion was waived by the ethics committee.

### 2.1. General Radiologists’ Cohort (GRC)

cMRI datasets were identified by text research in the Picture Archiving Communications System (PACS) and Radiology Information System (RIS) using search terms related to image acquisition time (December 2016 to April 2023), MRI scanner (1.5 Tesla MAGNETOM Avanto/Aera, Siemens Healthineers, Forchheim, Germany) and study descriptions indicating a cMRI including TOF-MRA (isotropic 1 mm voxel size including MIP reconstructions) acquired by the general radiology department. When repetitive cMRI scans of the same patient were identified, only the first acquired scan was included. No supplementary exclusion criteria were applied initially, finally yielding a dataset of 879 1.5T cMRI scans (879 different patients), all acquired and reported by the general radiology department.

### 2.2. Neuroradiologists’ Cohort (NRC)

For the purpose of data integration and comprehensive analysis, the cMRI study cohort from the previous pilot study by Schmidt et al. [18] was utilized, which included 907 cMRI datasets from 907 consecutive inpatients of the psychiatric department, examined on a 3.0T MRI scanner (MAGNETOM Prisma, Siemens Healthineers, Forchheim, Germany) with all of these cMRIs initially reported by neuroradiologists.

### 2.3. Data Management

**Figure 1:**
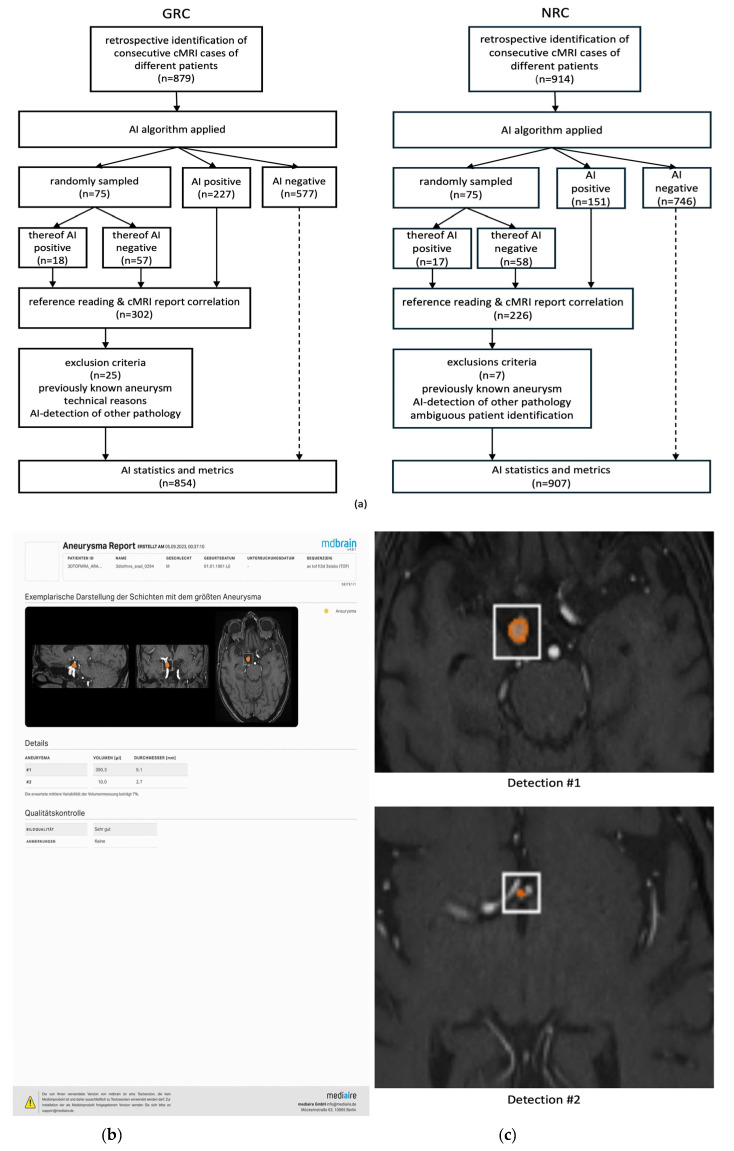
**Patient Enrollment Flowchart (GRC/NRC) and Illustration of AI Results.** Subfigure (**a**): The GRC flowchart (**left**) illustrates the AI analysis and selected reference readers’ reviews based on 879 initially included datasets, in addition to the equivalent flowchart of the NRC (**right**) previously established by Schmidt et al. [18]. Subfigure (**b**): AI reports provided after TOF-MRA analysis with a graphical illustration (for the largest detection) along with volumes and diameter quantifications (for all detections); a quality control of the underlying TOF-MRA sequence is provided. Subfigure (**c**): AI detections are highlighted by secondary captures in TOF-MRA duplicates. Abbreviations: AI—artificial intelligence; AI Positive—cMRIs with AI detections; AI Negative—cMRIs without AI detections; cMRI—cranial magnetic resonance imaging; GRC-general radiologist’s cohort; NRC—neuroradiologist’s cohort; TOF-MRA—time-of-flight magnetic resonance angiography.

DICOM export files of pseudonymized cMRI datasets were processed by a commercially available AI algorithm (see below). Following AI analysis, the pseudonymized cMRI datasets, including AI analysis reports (see Figure 1), were reintegrated into the PACS for the study-related reference reading (see below).

### 2.4. Artificial Intelligence Algorithm

The commercially available software mdbrain version 4.8 (Mediaire GmbH, Berlin, Germany), as previously introduced by Schmidt et al. [18], was implemented. The aneurysm segmentation algorithm consists of a 3D convolutional neural network with a U-NET architecture [29]. More than 400 cMRI datasets have been used for model training, including cMRIs of both healthy individuals and those with unruptured saccular cerebral aneurysms and including segmentations provided by an expert radiologist. Technical and algorithm training specifications have been described, e.g., by Schmidt et al. [18] and by Lehnen et al. [20]. Importantly, the training datasets did not include images from the institutions involved in the external algorithm validation of this study. A representative AI analysis report including quantitative metrics is illustrated in Figure 1.

### 2.5. Reference Reading and Analysis of Initial cMRI Reports

In both the general radiologists’ cohort (GRC) and the neuroradiologists’ cohort (NRC), 75 randomly selected cMRI datasets, along with all remaining datasets with AI detections (AI+), were reviewed by consultant-level neuroradiologists/neurointerventionalists. These reference readers were blinded to the initial cMRI reports and to all patient information, including sex and age (implying a significant remaining life expectancy). Reference readers were notably not involved in study design or cohort establishment. All originally acquired cMRI sequences, along with the illustrated AI results, were provided to the reference readers. AI results were displayed as secondary captures in TOF-MRA duplicates and presented as tabular data containing segmentation volumes and diameters (Figure 1b,c). Reference readers reviewed the cMRI datasets (including AI results) for intracranial aneurysms using Likert-based confidence scores for aneurysm suspicions as already introduced by the previous pilot study by Schmidt et al. [18]: 0—no aneurysm, 1—aneurysm unlikely, 2—aneurysm likely, 3—certain aneurysm. AI findings that were rejected by the reference readers (score 0) were further categorized based on whether they were localized at an intracranial artery or not. Suspicious findings (scores 1–3) were categorized based on location (ICA extradural, ICA infraclinoidal, ICA paraophthalmic, ICA supraophtalmic, Pcom origin, AchA origin, ICA bifurcation (“carotid T”/terminal ICA), MCA, ACA, Acom, other localization in the anterior circulation, V4 segment, PICA territory, basilar artery, basilar tip, SUCA origin, PCA territory, other localization in the posterior localization), morphology (saccular/fusiform/dysplastic-mixed), size (volumetry or maximal diameter), and aneurysm thrombosis (yes/no). Furthermore, reference readers provided recommendations regarding individual work-up strategies (“no consequence”/”non-invasive follow-up imaging, e.g., cMRI”/”further evaluation by catheter angiography [DSA]”).

A radiology resident and a well-trained medical student reviewed the initial cMRI reports. Information such as the original reason for cMRI imaging, reporting (neuro-)radiologists, and reported findings suspicious for intracranial aneurysms were extracted. Medical history was checked for previously known aneurysms. GRC and NRC characteristics are elucidated in Table 1. The study design and enrollment flowchart are illustrated in Figure 1a.

### 2.6. Patient Consultations

Based on the AI analysis, in conjunction with the study-related reference reading and assessment of initial cMRI reports, patients with newly identified findings suspicious for intracranial aneurysms were contacted and offered on-site neuroradiological consultations. Individual consultations included aneurysm rupture risk assessments (ISUIA [16]/UCAS [30]/PHASES [14] scoring) balanced with expected treatment risks (UIATS recommendations [13]) based on patient-specific factors, medical history and individual aneurysm characteristics. Individual work-up strategies involved cMRI follow-up imaging, additional catheter angiography (DSA) and interdisciplinary case reviews for recommended follow-up/therapy. When contact with patients was unsuccessful despite repeated attempts, aneurysm risk stratification was approximated using available information, such as aneurysm localization and size as derived from cMRI data, along with any available individual risk factors extractable from the medical records.

**Table 1:** **Study Cohort Characteristics (GRC and NRC).** Some cMRIs datasets have been excluded from statistical analysis due to technical reasons (e.g., missing initial reports; poor image quality (1×); and unclear detection numbering, predominantly in cases with AI detections not related to intracranial aneurysms), due to other AI-detected pathologies (AVM and intraventricular hemorrhage), and due to previously treated aneurysms or due to previously known intracranial aneurysms. The NRC as established by Schmidt et al. [18]. Abbreviations: GRC—general radiologist’s cohort; NRC—neuroradiologist’s cohort; AI—artificial intelligence; AI+ cMRI—cMRI with AI detections; cMRI—cranial magnetic resonance imaging; SAH—subarachnoid hemorrhage; AVM—arteriovenous malformation.

	GRC	NRC
cMRIs initially included/statistically analyzed [n]	879/854	914/907
cMRI acquisition period	9 December 2016—11 April 2023	21 October 2020—18 May 2022
patient age [mean ± standard deviation/median age]	59.8 ± 20.0 years/64 years	44.4 ± 20.5 years/45 years
MRI scanner	1.5 Tesla MAGNETOM Aera/Avanto (Siemens)	3 Tesla MAGNETOM Prisma (Siemens)
MR sequence used for AI analysis	Isotropic TOF-MRA, voxel size 1 mm	Isotropic TOF-MRA, voxel size 0.5mm
image quality evaluated by AI	767x “good” 111x “acceptable” 1x “rejected”	914x “good”
inclusion criteria for reference reading	75 randomly sampled cMRIs & all AI+ cMRIs (n = 302)	75 randomly sampled MRIs & all AI+ MRIs (n = 226)
**cMRI report characteristics**		
original reason for cMRI examination:		
○ direct question about intracranial aneurysms [n/%]	10/3.3%	7/3.1%
○ question about or related to circumstances that may be indirectly associated with possible intracranial aneurysms [n/%]	139/46.0%	56/24.8%
- acute or previous SAH [n/%]	11/3.6%	1/0.4%
- cranial nerve compression syndrome [n/%]	1/0.3%	1/0.4%
- any other vascular-related question [n/%]	115/38.1%	37/16.4%
- MRI before electroconvulsive therapy [n/%]	0/0.0%	11/4.9%
- headaches not suspicious for SAH [n/%]	12/4.0%	6/2.7%
○ question unrelated to intracranial aneurysm [n/%]	153/50.7%	163/72.1%
number of reporting radiologists involved [n]	83	8
**cMRIs excluded from statistical analysis**		
…due to previously known or treated aneurysms [n]	12	4
…due to technical reasons [n]	11	1
…due to AI detected other vasculopathies [n]	2	2

### 2.7. Statistics

Statistical analysis was focused on those cMRI findings suspicious for intracranial aneurysms that were not previously known and on patients previously unaware of intracranial aneurysms. A few cMRI datasets were excluded from analysis, e.g., due to previously known aneurysms, AI-detected vascular pathologies other than aneurysms, or technical issues (Table 1).

The AI algorithm performance was quantified by descriptive statistics and common diagnostic metrics. Aneurysm-size-based and localization-based subgroup analyses were performed. Reference readers’ Likert scores were converted into binary reference standards (RFS) as follows: The most specific RFS I focuses on findings rated as certain intracranial aneurysms (score 3) by pooling the other confidence scores 0–2 as negative for intracranial aneurysms. On the contrary, the most sensitive RFS III (“rule out scenario”) focuses on all at least suspicious findings by pooling the scores 1–3 as positive for intracranial aneurysms. The intermediate RFS II (scores 2/3 rated as positive for aneurysms) was computed accordingly. To quantify the added value of an AI screening, suspicious findings were correlated with the initial cMRI reports. By reviewing the AI results in conjunction with the reference reading, along with the initial cMRI reports, the number needed to screen (NNS) was defined as the number of cMRIs requiring AI-based review to identify one additional clinically relevant aneurysm or to trigger one follow-up imaging.

## 3. Results

The GRC results are initially presented in detail, in each paragraph, followed by a comparison to the NRC results previously reported in detail by Schmidt et al. [18]. A statistical synthesis of the GRC and NRC results is finally provided for a focused holistic analysis.

### 3.1. GRC Characteristics

GRC patient’s mean age was 59.8 ± 20.0 years with a median of 64 years (Table 1). The AI algorithm classified the TOF-MRA image quality as good in 767 GRC cases (87.26%) and acceptable in 111 GRC cases (12.63%) and rejected one of the initially enrolled datasets (0.11%); see Table 1. The initial cMRI requests were extracted for all 302 GRC patients within the reference reading; notably, 96.7% of these cMRIs were performed without any direct question about intracranial aneurysms. GRC result analysis was finally based on 854 cMRI datasets (854/879 = 97.2%, for exclusion criteria, see Table 1).

Compared to the NRC [18], the GRC patients were older (median 64 years [GRC] vs. 45 years [NRC]), the quality of 1.5T GRC cMRI datasets was lower (“good” in 87.3% [GRC] vs. 100% [NRC]) and a comparably estimated proportion of original cMRI requests were not directly related to questions about aneurysms (96.7% [GRC] vs. 96.9% [NRC]); see Table 5.

### 3.2. GRC—AI Algorithm Performance

Based on the finally analyzed cohort of 854 GRC cMRI scans, the AI algorithm detected 314 findings in 226 different cMRI datasets (Table 2), resulting in an alert rate of 26.5% (226/854). This detection rate surpasses the estimated prevalences of intracranial aneurysms in the study cohort, which ranged from 1.9% (16/854, RFS1) to 7.7% [(16 + 16 + 34)/854, RFS3], depending on the consideration of inconclusive findings (Table 2). The reference readers rejected 79.0% (111 + 137/314) of the AI-detections as not suspicious for an intracranial aneurysm (score = 0), with a notable subset (n = 111) without any association with an artery (Table 2 and Table 3). The resulting high false positive rates (FPRs) of the AI algorithm ranged between 79.0% [(137 + 111)/314, RFS1] and 94.9% [(314-16)/314, RFS3] depending on the applied RFS (Table 2 and Table 3).

As for the overall positive predictive values (PPVs) of the AI algorithm, reference readers considered an underlying aneurysm (scores 1–3) in 21.0% (66/314) of all AI detections, which represents the most sensitive RFS interpretation (Table 2 and Table 4). PPVs decreased to 10.2% [(16 + 16)/314] and 5.1% (16/314) in the case of considering only AI findings with underlying intracranial aneurysms regarded as at least probable (scores 2 + 3) or certain (score 3); see Table 2 and Table 4. A majority, 93.6% (15/16), of score 3 findings exceeded 2 mm in diameter (Table 3). On the contrary, a majority, 86.7% (67 + 52/137), of the artery-associated score 0 findings were measured with diameters less than 4 mm (Table 3). This finally results in PPVs depending on the detection sizes: PPVs of 0.8–13.2% were achieved for findings smaller than 2 mm in diameter, and the PPVs increased to a range of 18.8–31.3% for findings of 4–6 mm in diameter (Table 4).

Regarding sensitivity quantification, the algorithm demonstrated 100% sensitivity for findings of any suspicion that exceed 6 mm in diameter as well as for smaller findings exceeding 4 mm in diameter if considering only detections that have been rated as at least likely aneurysms (scores 2/3); see Table 4. One reference readers’ score 3 finding (2.7 mm in diameter), two reference readers’ score 2 findings (2.8/3.3 mm in diameter), and two reference readers’ score 1 findings (2.0/5.6 mm in diameter) were missed by the AI algorithm (Table 2 and Table 3). Notably, only the score 3 finding (2.7 mm in diameter) was documented in the initial cMRI report. The resulting overall sensitivities remain well above 90%, regardless of which findings of which suspicion grades (scores 1–3) were finally considered for sensitivity calculation (Table 4).

Comparing GRC with NRC results [18], there was a higher AI alert rate in the GRC (26.5% [GRC] vs. 17.8% [NRC]) despite of comparable aneurysm prevalences (1.9–7.7% [GRC] vs. 2.3–6.5% [NRC]), in this context explaining higher FPRs (79.0–94.9% [GRC] vs. 67.6–88.5% [NRC]) and lower PPVs (5.1–21.0% [GRC] vs. 11.5–32.4% [NRC]); see Table 5. AI sensitivities were roughly comparable in both cohorts: 94.1% [GRC] vs. 100% [NRC] sensitivity for certain aneurysms of any size and >90.9% [GRC] vs. 100% [NRC] sensitivity also for less suspicious findings exceeding 4 mm in diameter.

### 3.3. GRC—Clinical Impact of an AI-Based Routine Screening

**Figure 2:**
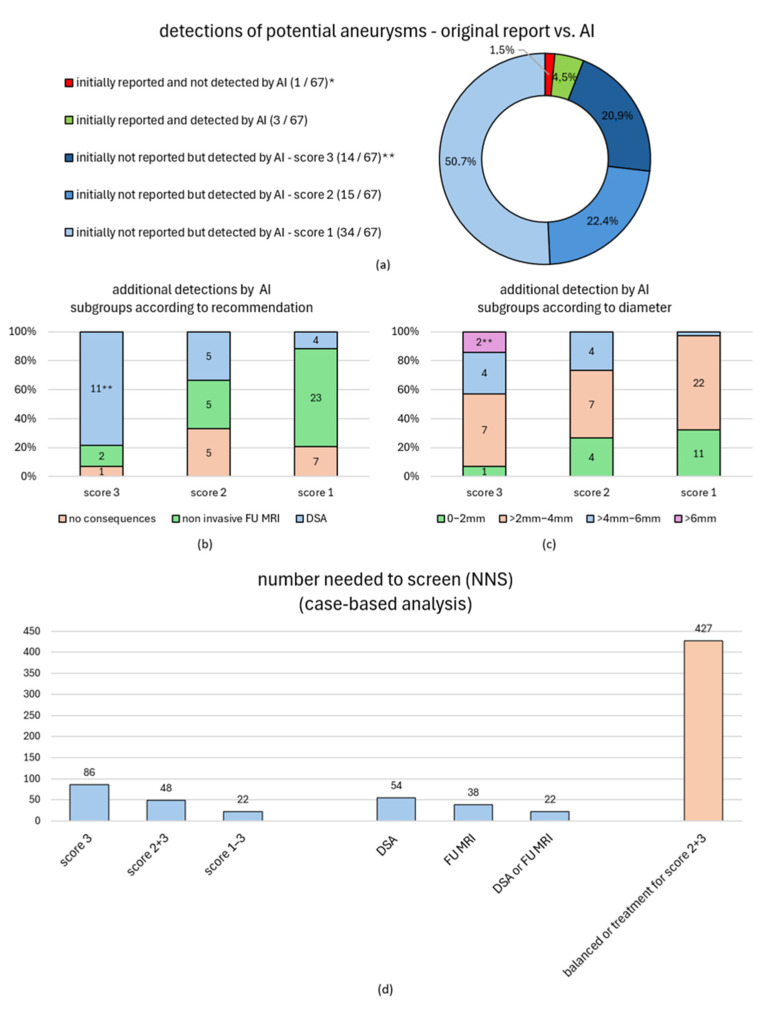
**General Radiologists’ Cohort (GRC)—Graphical Overview of Findings Additionally Detected by AI Additional (a–c) and a Case-Based NNS Analysis to Quantify the Impact of AI-Based Routine cMRI Screening (d)**. Subfigure (**a**): AI-based detections suspicious for aneurysms (scores 1–3) as opposed to the initially reported findings. * One score 3 MCA finding MCA was not detected by the AI. ** One of these fourteen detections was described by the initial report but not assessed as an aneurysm; the reference readers recommended a DSA work-up. Subfigure (**b**): Work-up recommendations based on additionally uncovered findings. All findings without any work-up recommendation refer to extradural ICA aneurysms. Subfigure (**c**): Diameter categories of additionally detected findings. Subfigure (**d**): The cMRI dataset quantity requiring screening (NNS) to additionally uncover one finding varying confidence and/or of clinical significance as outlined above. The NNS calculation focused on a possible therapeutic impact (orange bar) based on UIAT score estimations according to Figure 3. An analogous analysis for the NRC findings can be reviewed in Schmidt et al. [18]. Abbreviations: AI—artificial intelligence; cMRI—cranial magnetic resonance imaging; DSA—digital subtraction angiography; FU MRI—follow-up magnetic resonance imaging; MCA—middle cerebral artery; NNS—number needed to screen; UIAT score—unruptured intracranial aneurysm treatment score.

**Figure 3:**
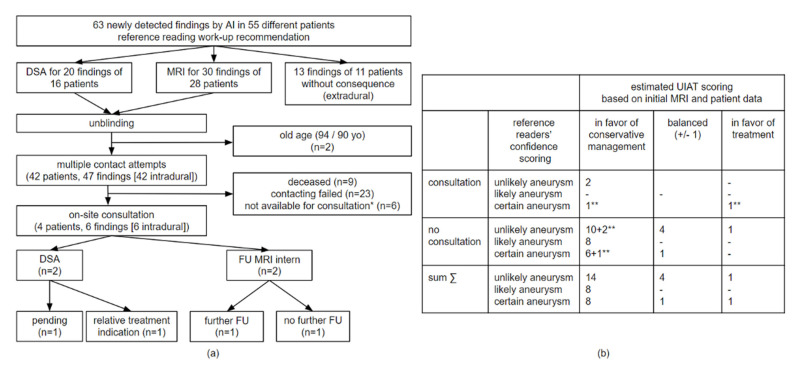
**General Radiologists’ Cohort (GRC)-Patient Enrollment for On-Site Consultations (a) and UIAT Scoring of Additionally Uncovered and Suspicious Findings.** Subfigure (**a**): Flowchart illustrating the progression from reference readers’ blinded work-up recommendations to unblinded on-site consultations including further imaging acquisition, ultimately leading to individualized work-up strategies (only suspicious findings newly uncovered through the study-related AI-screening considered). * Patients were contacted but not available for on-site consultation (FU cMRI and consultation on other occasions recommended). Subfigure (**b**): UIAT scoring based on cMRIs and patient data/risk factors as assessed by consultations or by reviewing existing medical records. UIAT scoring with differences ±1 were regarded as balanced as per the definition, in favor of treatment/conservative management accordingly. Illustrated results refer to datasets (not to findings); NNS calculations (Figure 2d) are based on these quantifications. ** Patients with more than one finding were classified according to the most relevant one. The same and similarly presented analysis for the NRC can be found in Schmidt et al. [18]. Abbreviations: AI—artificial intelligence; DSA—digital subtraction angiography; FU MRI— follow-up magnetic resonance imaging; UIAT score—unruptured intracranial aneurysm treatment score; yo—years old.

A potential benefit of introducing a second-reader routine screening depends on several factors, including the performance of the AI algorithm, the baseline accuracy of the reporting (neuro-) radiologists without AI assistance, and the clinical significance of the findings additionally identified by an AI second-reader screening. The initial cMRI reports missed 94.4% (67/71) of possible intracranial aneurysms (scores 1–3) as defined by the reference reading (Table 2). The AI-algorithm, on the other hand, detected 93,0% (66/71) of suspicious findings (Table 2). One out of the five reference readers’ cMRI findings missed by the AI algorithm was described in the original cMRI report (Table 2). Summing up, the AI-tool detected 94.0% (63/67) of the initially non-reported findings suspicious for an intracranial aneurysm, which were assessed by the reference readers as follows (see Table 2): 14 certain aneurysms (score 3, 11 intradural), 15 likely aneurysms (score 2, 9 intradural), and 34 unlikely aneurysms (score 1, 25 intradural); see Table 2 and Table 3 and Figure 2a–c. The majority of newly identified potential aneurysms ranged between 2 mm and 4 mm in diameter (Table 3, Figure 2c). The reference readers’ follow-up recommendations for intradural findings were as follows: additional DSA in 20 cases and cMRI follow-up in 30 cases (spread over 44 different patients); no follow-up recommendations for the other additional 13 extradural findings (Figure 2b and Figure 3a).

In the case of newly detected aneurysm-suspicious findings with a follow-/work-up recommendation, multiple attempts were made to contact the patients (except for two elderly patients [94/90 years old]). There was a high fall-out rate and only 4 out of 42 patients (9.5%) were available for an individual on-site consultation (Figure 3a). Another follow-up cMRI was acquired in two cases and an additional DSA was planned for the other two cases with one of them already performed and resulting in an offer for aneurysm treatment.

With an exclusive focus on intradural localization, the AI additionally detected 42 suspicious findings in 37 different patients, resulting in numbers of cMRIs that needed to be screened (NNS) as follows (Figure 2d). For the additional detection of one intradural score 3 (“certain”)/score 2 + 3 (“at least likely”)/score 1–3 (“any possible”) finding, the associated NNSs were 86/48/22. Based on the reference readers’ workup recommendations, the NNS of 22 consequently also refers to any additional imaging (cMRI or DSA) with a higher NNS of 54 for directly indicating a DSA workup. UIAT scores were estimated for suspicious intradural findings based on available data to estimate the possible therapeutic impact (Figure 3b): The NNS to additionally uncover one at least probable aneurysm (scores 2 + 3) associated with a UIAT scoring at least balanced (or favoring treatment) was 427 (Figure 2d).

**Table 2:** **General Radiologists’ Cohort (GRC)—Detection-Based Descriptive Statistics.** Statistics of 319 cMRI detections suspicious for intracranial aneurysms across 226 different cMRI datasets. The findings were extracted from 854 cMRI datasets, which were screened by the AI algorithm, along with a neuroradiologist’s reference reading of all AI+ cMRI datasets and an initial sample of 75 randomly selected datasets (Figure 1a). Reference readers classified suspicious cMRI findings using Likert-based confidence scores *. Categorial work-up recommendations included “no consequences”, “non-invasive FU MRI” and “DSA”. All suspicious findings without work-up recommendations were extradural ICA aneurysms. The same and similarly presented analysis for the NRC can be found in Schmidt et al. [18]. Abbreviations: AI—artificial intelligence; AI+—cMRIs with AI detections; cMRI—cranial magnetic resonance imaging; FU MRI—follow-up magnetic resonance imaging; DSA—digital subtraction angiography; RFS—reference standard; GRC—general radiologists’ cohort; NRC—neuroradiologists’ cohort.

319 cMRI Findings Suspicious for Intracranial Aneurysms as Detected by the AI Algorithm and/or the Neuroradiologists’ Reference Reading.									
	Reading Score *	Detections [n (%)]	Thereof Initially not Reported [n (%)]	Recommendations (Reference Reading)	Detections [n (%)]	Thereof Initially Not Reported [n (%)]	Prevalence Within RFS [n (%)]		
							RFS I **	RFS II **	RFS III **
detected by AI	score 3 * [n (%)]	16/319 (5.0%)	14/16 (87.5%)	no consequences	1/16 (6.3%)	1/1 (100%)	16/854 (1.9%)	32/854 (3.7%)	66/854 (7.7%)
				non-invasive FU MRI	2/16 (12.5%)	2/2 (100%)			
				DSA	13/16 (81.3%)	11/13 (84.6%)			
	score 2 * [n (%)]	16/319 (5.0%)	15/16 (93.8%)	no consequences	5/16 (31.3%)	5/5 (100%)			
				non-invasive FU MRI	5/16 (31.3%)	5/5 (100%)			
				DSA	6/16 (37.5%)	5/6 (83.3%)			
	score 1 * [n (%)]	34/319 (10.7%)	34/34 (100%)	no consequences	7/34 (20.6%)	7/7 (100%)			
				non-invasive FU MRI	23/34 (67.6%)	23/23 (100%)			
				DSA	4/34 (11.8%)	4/4 (100%)			
	score 0 *, associated with intracranial artery [n (%)]	137/319 (42.9%)	137/137 (100%)	no consequences	137/137 (100%)	137/137 (100%)			
				non-invasive FU MRI	0/137 (0%)	-			
				DSA	0/137 (0%)	-			
	score 0 *, not associated with intracranial artery [n (%)]	111/319 (34.8%)	111/111 (100%)	no consequences	111/111 (100%)	111/111 (100%)			
				non-invasive FU MRI	0/111 (0%)	-			
				DSA	0/111 (0%)	-			
not detected by AI	score 3 *, not detected by AI [n (%)]	1/319 (0.3%)	0/1 (0%)	no consequences	0/1 (0%)	-			
				non-invasive FU MRI	0/1 (0%)	-			
				DSA	1/1 (100%)	0/1 (0%)			
	score 2 *, not detected by AI [n (%)]	2/319 (0.6%)	2/2 (100%)	no consequences	0/2 (0%)	-			
				non-invasive FU MRI	0/2 (0%)	-			
				DSA	2/2 (100%)	2/2 (100%)			
	score 1 *, not detected by AI [n (%)]	2/319 (0.6%)	2/2 (100%)	no consequences	0/2 (0%)	-			
				non-invasive FU MRI	2/2 (100%)	2/2 (100%)			
				DSA	0/2 (0%)	-			
* Likert-based reference reading confidence scores: 0—no aneurysm (AI detection further assessed based on the position relative to intracranial arteries), 1—aneurysm unlikely, 2—aneurysm likely, 3—certain aneurysm. ** Prevalences within the study cohort are calculated based on three differentially sensitive reference standards: The most specific RFS I only considers the reference reading score 3 as positive for an aneurysm, the most sensitive RFS III pools the reference reading scores 1–3 as positive for an aneurysm. The intermediate RFS II considers the reference reading scores 2/3 as positive for an aneurysm.									

**Table 3:** **General Radiologists’ Cohort (GRC)—Detection-Based Statistics and Subgroup Analysis (Aneurysm Localization and Size).** In addition to Table 2, a subgroup analysis based on detection sizes (volume/diameter) and localization categories ** is illustrated. The same and similarly presented analysis for the NRC can be found in Schmidt et al. [18]. Abbreviations: AI—artificial intelligence; ICA—internal carotid artery; AcomA—anterior communicating artery; ACA—anterior cerebral artery; MCA—middle cerebral artery; PICA—posterior inferior cerebellar artery; SCA—superior cerebellar artery; PCA—posterior cerebral artery; Pcom—Posterior communicating artery; AchA—anterior choroidal artery; GRC—general radiologists’ cohort; NRC—neuroradiologists’ cohort.

General Radiologists’ Cohort (GRC)—Detection-Based Statistics and Subgroup Analysis (Aneurysm Localization and Size)									
	Reading Score	Diameter [mm]	Aneurysm [n(%)]	Thereof Initially Not Reported [n(%)]	Volume [mL]	Aneurysm [n(%)]	Thereof Initially Not Reported [n(%)]	Localization **	Thereof Initially Not Reported [n(%)]
detected by AI	score 3 * [n(%)]	0–2 mm >2–4 mm >4–6 mm >6 mm	1/16 (6.3%) 7/16 (43.8%) 5/16 (31.3%) 3/16 (18.8%)	1/1 (100%) 7/7 (100%) 4/5 (80.0%) 2/3 (66.7%)	0–5 mL >5 mL–15 mL >15 ml–30 mL >30 mL	2/16 (12.5%) 2/16 (12.5%) 4/16 (25.0%) 8/16 (50.0%)	2/2 (100%) 2/2 (100%) 4/4 (100%) 6/8 (75.0%)	anterior 5/16 (31.3%) posterior 2/16 (12.5%) ICA intradural 6/16 (37.5%) ICA extradural 3/16 (18.8%)	3/5 (60.0%) 2/2 (100%) 6/6 (100%) 3/3 (100%)
	score 2 * [n(%)]	0–2 mm >2–4 mm >4–6 mm >6 mm	4/16 (25.0%) 8/16 (50.0%) 4/16 (25.0%) 0/16 (0%)	4/4 (100%) 7/8 (87.5%) 4/4 (100%) -	0–5 mL >5 mL–15 mL >15 mL–30 mL >30 mL	5/16 31.3% 4/16 25.0% 3/16 (18.8%) 4/16 (25.0%)	5/5 (100%) 4/4 (100%) 2/3 (66.7%) 4/4 (100%)	anterior 7/16 (43.8%) posterior 2/16 (12.5%) ICA intradural 1/16 (6.3%) ICA extradural 6/16 (37.5%)	6/7 (85.7%) 2/2 (100%) 1/1 (100%) 6/6 (100%)
	score 1 * [n(%)]	0–2 mm >2–4 mm >4–6 mm >6 mm	11/34 (32.4%) 22/34 (64.7%) 1/34 (2.9%) 0/34 (0%)	11/11 (100%) 22/22 (100%) 1/1 (100%) -	0–5 mL >5 mL–15 mL >15 mL–30 mL >30 mL	12/34 (35.3%) 14/34 (41.2%) 6/34 (17.6%) 2/34 (5.9%)	12/12 (100%) 14/14 (100%) 6/6 (100%) 2/2 (100%)	anterior 11/34 (32.4%) posterior 4/34 (11.8%) ICA intradural 10/34 (29.4%) ICA extradural 9/34 (26.5%)	11/11 (100%) 4/4 (100%) 10/10 (100%) 9/9 (100%)
	score 0 *, associated with intracranial artery [n(%)]	0–2 mm >2–4 mm >4–6 mm >6 mm	67/137 (48.9%) 52/137 (38.0%) 9/137 (6.6%) 9/137 (6.6%)	67/67 (100%) 52/52 (100%) 9/9 (100%) 9/9 (100%)	0–5 mL >5 mL–15 mL >15 mL–30 mL >30 mL	70/137 (51.1%) 41/137 (29.9%) 8/137 (5.8%) 18/137 (13.1%)	70/70 (100%) 41/41 (100%) 8/8 (100%) 18/18 (100%)	anterior 23/137 (16.8%) posterior 6/137 (4.4%) ICA intradural 19/137 (13.9%) ICA extradural 28/137 (20.4%) not specified 61/137 (44.5%)	23/23 (100%) 6/6 (100%) 19/19 (100%) 28/28 (100%) 61/61 (100%)
	score 0 *, not associated with intracranial artery [n(%)]	0–2 mm >2–4 mm >4–6 mm >6 mm	38/111 (34.2%) 41/111 (36.9%) 13/111 (11.7%) 19/111 (17.1%)	38/38 (100%) 41/41 (100%) 13/13 (100%) 19/19 (100%)	0–5 mL >5 mL–15 mL >15 mL–30 mL >30 mL	38/111 (34.2%) 25/111 (22.5%) 15/111 (13.5%) 34/111 (30.6%)	38/38 (100%) 25/25 (100%) 15/15 (100%) 34/34 (100%)	-	-
not detected by AI	score 3 *, undetected by AI [n(%)]	0–2 mm >2–4 mm >4–6 mm >6 mm	0/1 (0%) 1/1 (100%) 0/1 (0%) 0/1 (0%)	- 0/1 (0%) - -	0–5 mL >5 mL–15 mL >15 mL–30 mL >30 mL		***	anterior 1/1 (100%) posterior 0/1 (0%) ICA intradural 0/1 (0%) ICA extradural 0/1 (0%)	0/1 (0%) - - -
	score 2 *, undetected by AI [n(%)]	0–2 mm >2–4 mm >4–6 mm >6 mm	0/2 (0%) 2/2 (100%) 0/2 (0%) 0/2 (0%)	- 2/2 (100%) - -	0–5 mL >5 mL–15 mL >15 mL–30 mL >30 mL			anterior 0/2 (0%) posterior 2/2 (100%) ICA intradural 0/2 (0%) ICA extradural 0/2 (0%)	- 2/2 (100%) - -
	score 1 *, undetected by AI [n(%)]	0–2 mm >2–4 mm >4–6 mm >6 mm	0/2 (0%) 1/2 (50.0%) 1/2 (50.0%) 0/2 (0%)	- 1/1 (100%) 1/1 (100%) -	0–5 mL >5 mL–15 mL >15 mL–30 mL >30 mL			anterior 1/2 (50.0%) posterior 1/2 (50.0%) ICA intradural 0/2 (0%) ICA extradural 0/2 (0%)	1/1 (100%) 1/1 (100%) - -
* Likert-based reference reading confidence scores see caption Table 2. ** anterior cerebral circulation (AcomA, ACA, MCA, other arteries anterior circulation), posterior cerebral circulation (basilar artery, V4 segment, PICA, SCA, PCA, other arteries posterior circulation), ICA intradural (including terminal ICA, Pcom, AchA), ICA extradural. *** AI-based volumetry not available (findings not detected by AI).									

When comparing these aspects to the NRC results [18], initial cMRI reporting by (neuro)radiologists demonstrated comparably high risks of incidental aneurysm non-reporting, with rates of 94.4% within the GRC and 86.4% within the NRC (Table 5). Regarding the impact of AI screening, the NNS for recommending follow-up imaging based on reference readers’ recommendations was lower in the GRC (22 [GRC] vs. 26 [NRC]). However, the NNS for detecting highly suspicious findings (scores 2 + 3) with potential treatment implications was lower in the NRC (427 [GRC] vs. 152 [NRC]), with the lower median age of NRC patients in better physical condition potentially contributing to this (UIATS-related) difference (Table 5).

**Table 4:** **Detection-Based Statistics (PPV / Sensitivity) Including Size-Dependent Subgroup Analysis**. Sensitivities and positive predictive values (PPVs) were quantified for differentially sensitive reference standards (see caption of Table 2) as an overall analysis as well as in a size-dependent subgroup analysis. PPVs and sensitivities are differentially calculated for the GRC (yellow), the NRC [18] (red), and the synthesized cohort (green). Abbreviations: AI—artificial intelligence; PPV—positive predictive value; RFS—reference standard; GRC—general radiologists’ cohort; NRC—neuroradiologists’ cohort.

			%(n)		
			RFS I	RFS II	RFS III
**Overall Analysis**					
PPV		GRC (all = 314)	5.1% (16/314)	10.2% (32/314)	21.0% (66/314)
		NRC (all =182)	11.5% (21/182)	19.7% (36/182)	32.4% (59/182)
		Synthesized	7.5% (37/496)	13.7% (68/496)	25.2% (125/496)
Sensitivity		GRC (all = 319)	94.1% (16/17)	91.4% (32/35)	93.0% (66/71)
		NRC (all=189)	100% (21/21)	83.7% (36/43)	89.4% (59/66)
		Synthesized	97.4% (37/38)	87.2% (68/78)	91.2% (125/137)
**Subgroup Analysis (Diameter)**					
0–2 mm	PPV	GRC	0.8% (1/121)	4.1% (5/121)	13.2% (16/121)
		NRC	2.3% (2/88)	5.7 (5/88)	15.9% (14/88)
		Synthesized	1.4% (3/209)	4.8% (10/209)	14.4% (30/209)
	Sensitivity	GRC	100% (1/1)	100% (5/5)	100% (16/16)
		NRC	100% (2/2)	55.6% (5/9)	77.7% (14/18)
		Synthesized	100% (3/3)	71.4% (10/14)	88.2% (30 /34)
>2–4 mm	PPV	GRC	5.4% (7/130)	11.5% (15/130)	28.5% (37/130)
		NRC	17.1% (12/70)	31.4% (22/70)	51.4% (36/70)
		Synthesized	9.5% (19/200)	18.5% (37/200)	36.5% (73/200)
	Sensitivity	GRC	87.5% (7/8)	83.3% (15/18)	90.2% (37/41)
		NRC	100% (12/12)	88.0% (22/25)	92.3% (36/39)
		Synthesized	95.0% (19/20)	86.0% (37/43)	91.3% (73/80)
>4–6 mm	PPV	GRC	18.8% (6/32)	31.3% (10/32)	31.3% (10/32)
		NRC	43.8% (7/16)	43.8% (7/16)	43.8% (7/16)
		Synthesized	27.1% (13/48)	35.4% (17/48)	35.4% (17/48)
	Sensitivity	GRC	100% (6/6)	100% (10/10)	90.9% (10/11)
		NRC	100% (7/7)	100% (7/7)	100% (7/7)
		Synthesized	100% (13/13)	100% (17/17)	94.4% (17/18)
>6 mm	PPV	GRC	9.7% (3/31)	9.7% (3/31)	9.7% (3/31)
		NRC	0% (0/8)	0% (0/8)	0% (0/8)
		Synthesized	7.7% (3/39)	7.7% (3/39)	7.7% (3/39)
	Sensitivity	GRC	100% (3/3)	100% (3/3)	100% (3/3)
		NRC	-	-	-
		Synthesized	100% (3/3)	100% (3/3)	100% (3/3)

### 3.4. Cohort Synthesis (GRC and NRC) and Holistic Analysis

**Figure 4:**
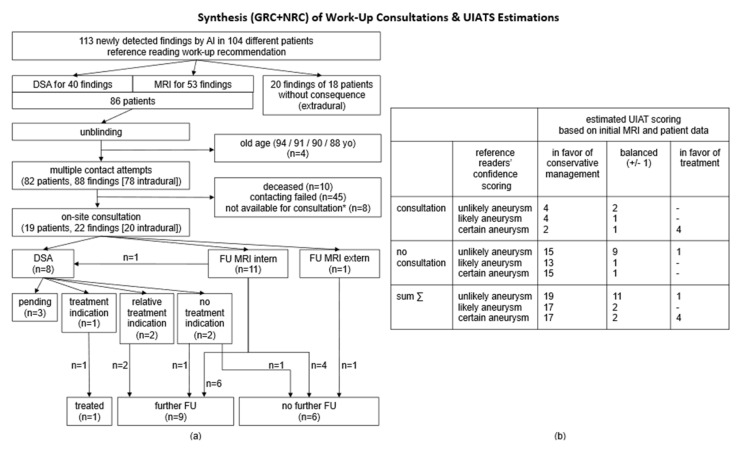
**Holistic Analysis of Synthesized Cohort (GRC + NRC [18])—Enrollment Flowchart for On-Site Consultations (a) and UIAT Score Estimations (b).** Subfigure (**a**): Flowchart illustration according to Figure 3a but based on the synthesized cohort (GRC + NRC). Subfigure (**b**): UIAT score estimations according to Figure 3b but referring to the synthesized overall cohort (GRC + NRC). Abbreviations: AI—artificial intelligence; DSA— digital subtraction angiography; FU MRI—follow-up magnetic resonance imaging; GRC—general radiologist’s cohort; NRC—neuroradiological cohort; UIAT score—unruptured intracranial aneurysm treatment score; yo—years old. * Patients were contacted but not available for on-site consultation (FU cMRI and consultation on other occasions recommended).

**Figure 5:**
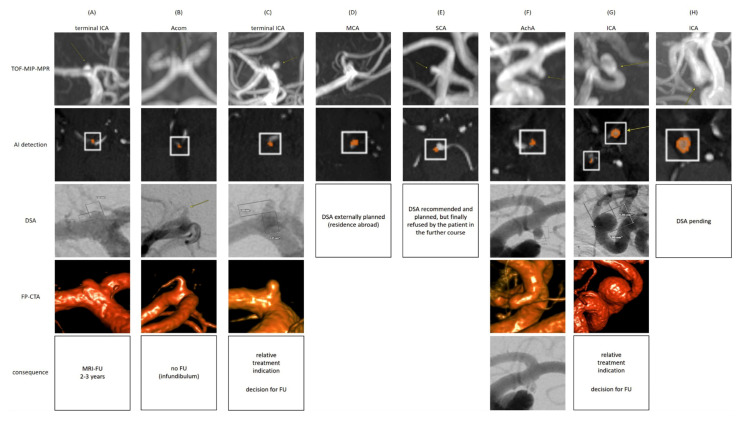
**DSA Work-Ups Due to Newly Uncovered Findings Suspicious for Intradural Aneurysms.**Subfigure (**A**): A 55-year-old female with a suspected terminal ICA aneurysm who preferred DSA over cMRI FU, ultimately uncovering an initial aneurysm (1–2 mm); no treatment indication; FU cMRIs at a multi-year interval were scheduled. Subfigure (**B**): A 66-year-old female with an inconclusive TOF-MRA Acom finding who favored DSA over FU cMRI after consultation; an infundibulum was uncovered without any work-up/follow-up required. Subfigure (**C**): A 32-year-old male with a terminal ICA aneurysm (max. 2 mm) as confirmed by DSA; relative treatment indication according to neurosurgical/neuroradiological evaluation; final decision for cMRI FU at a multi-year interval and risk factor adjustment (stop smoking). Subfigure (**D**): A 38-year-old male with a suspected MCA aneurysm was scheduled for DSA (finally planned externally for patient-specific reasons). Subfigure (**E**): A 61-year-old female with a suspected SUCA aneurysm was scheduled for catheter angiography that was refused by the patient in the further course in favor of cMRI follow-up. Subfigure (**F**): A 58-year-old female with a saccular AchA aneurysm max. approx. 3.4 × 1.8 mm (dome height × neck width, see DSA); aneurysm coil occlusion was performed without any complications. Subfigure (**G**): A 22-year-old male with an ICA aneurysm max. approx. 6.1 × 5.8 mm presumably in the context of an underlying Alagille Syndrome. The aneurysm did not change in size over the past four years. An additionally acquired cMRI demonstrated a primarily suspected extradural localization (as far as evaluable) with cranialization of the dura by the aneurysm. In the case of a relative treatment indication, a decision for a cMRI follow-up was made. Subfigure (**H**): A 63-year-old male with a mixed sacciform–fusiform ICA aneurysm max. approx. 3.8 × 6.9 mm (dome height × neck width), a history of liver transplantation, and currently a suspicion of malignancy under evaluation; the DSA is pending. Subfigures (**A**–**F**) are reproduced/taken from the preceding study by Schmidt et al. [18]. Abbreviations: f—female; m—male; yo—years old; ICA—internal carotid artery; Acom—anterior communicating artery; MCA—middle cerebral artery; SCA—superior cerebellar artery; AchA—anterior choroidal artery; TOF-MIP-MPR—time-of-flight maximum-intensity-projection multi-planar reconstruction; AI—artificial intelligence; DSA—digital subtraction angiography; FP-CTA—flat-panel computed tomography angiography; FU—follow-up.

Considering the synthesized cohort (GRC + NRC) including 1761 cMRI scans altogether, the overall aneurysm prevalence ranged from 2.1% for the least inclusive RFS up to 7.1% for the most inclusive RFS (Table 5). The majority, 90.5% (124/137), of suspicious findings were not mentioned in the initial cMRI reports. The AI algorithm led to a cMRI alert rate of 22.0% (387/1761), in a detection-based analysis associated with PPVs of 7.5% (37/496, RFS3) up to 25.2% (125/496, RFS1) and FPRs of 61.9% (307/496, RFS1) up to 92.5% (459/496, RFS3), depending on the applied RFS’s sensitivity (Table 4). Overall algorithm sensitivities for the detection of certain aneurysms/any suspicious findings were 97.4% (37/38)/91.2% (125/137); see Table 4 and Table 5. Regarding NNS quantifications of intradural findings, 77/24 cMRIs had to be screened to additionally uncover one certain aneurysm (score 3)/one finding of any suspicion (scores 1–3); an NNS of 24 was consequently necessary to indicate any kind of work-/follow-up (cMRI or DSA). Taking the on-site consultations and UIAT score estimations (Figure 4b) into account, an NNS of 221 was necessary to additionally detect one finding likely corresponding to an intradural aneurysm (scores 2/3) with scoring at least balanced or favoring treatment (Table 5). Moreover, patient contact attempts, on-site consultations, and resulting follow-/work-up strategies are illustrated in Figure 4b. As derived from the limited consultation rate of 23% (19 out of 82 initially contacted patients), twelve follow-up cMRIs and eight DSAs (one patient underwent both follow-up cMRI and DSA) were initiated (example images shown in Figure 5), ultimately resulting in three cases with at least a relative indication for treatment and another seven cases without treatment indication but requirement for further follow-ups at multi-year intervals. Based on the synthesized cohort size of 1761 cMRI scans, the extrapolation to a 100% consultation rate (using a factor of 100/23 = 4.35) would suggest the following outcomes: 52 patients (2.95%) would undergo at least one follow-up cMRI, 35 patients (1.99%) would undergo diagnostic DSA, and 13 patients (0.74%) would have an additionally detected aneurysm with an at least relative indication for treatment. These post-consultation estimates are slightly higher than those derived from NNS quantifications based on the reference reading alone (Table 5), which are as follows: 2.17% (NNS 48) of patients with at least one follow-up cMRI, 2.04% (NNS 49) of patients with the recommendation for diagnostic DSA, and 0.45% (NNS 221) of patients with a relevant finding and possible therapeutic impact.

**Table 5:** **Holistic Analysis of Synthesized Cohort (GRC + NRC [18])—Comparative Cohort Parameters and Key Results.** Abbreviations: AI—artificial intelligence; PPV—positive predictive value; FPR—false positive rate; RFS—reference standard; GRC—general radiologists’ cohort; NRC—neuroradiologists’ cohort; DSA—digital subtraction (catheter) angiography; UIATS—unruptured intracranial aneurysm treatment score; FU—follow-up.

	GRC	NRC	Synthesized
age [mean ± standard deviation/median]	59.8 ± 20.0 years/64 years	44.4 ± 20.5 years/45 years	51.9 ± 21.7 years/54 years
aneurysm prevalence (depending on applied RFSs)	1.9–7.7%	2.3–6.5%	2.1–7.1%
findings score 0/1/2/3 [n]	148/34/16/16	123/23/15/21	271/57/31/37
diameter [mean ± standard deviation/median] score 1 findings score 2 findings score 3 findings	2.4 ± 0.8 mm/2.4 mm 2.9 ± 1.2 mm/2.5 mm 4.3 ± 2.1 mm/4.0 mm	2.2 ± 0.9 mm/2.3 mm 2.5 ± 0.7 mm/2.6 mm 3.5 ± 1.2 mm/3.3 mm	2.3 ± 0.8 mm/2.3 mm 2.7 ± 1.0 mm/2.6 mm 3.9 ± 1.7 mm/3.5 mm
initially non-reported findings (scores 1–3) [%]	94.4%	86.4%	90.5%
**AI performance (detection-based analysis)**			
AI cMRI alert rate [%]	26.5%	17.8%	22.0%
100% AI detection sensitivity of certain aneurysm	>2.7 mm	any size	>2.7 mm
algorithm sensitivity for the detection of certain aneurysms/any suspicious finding [%]	94.1%/93.0%	100%/89.4%	97.4%/91.2%
PPV range depending on applied RFS sensitivity [%]	5.1–21.0%	11.5–32.4%	7.5–25.2%
FPR range depending on applied RFS sensitivity [%]	79.0–94.9%	67.6–88.5%	61.9–92.5%
**NNS (case-based analysis)**			
certain aneurysm (score 3) [n]	86	70	77
any suspicious finding (scores 1–3) [n]	22	26	24
recommended FU MRI [n]	38	57	48
recommended DSA [n]	54	46	49
recommended DSA or FU MRI [n]	22	26	24
UIATS balanced or treatment for scores 2/3 [n]	427	152	221

## 4. Discussion

The presented study externally validated a commercial AI solution for aneurysm detection in TOF-MRAs using a representative cohort, comprising both a newly established general radiologists’ 1.5T dataset (GRC, representative inpatient cohort of a maximum-care university hospital) and a previously established neuroradiologists’ 3.0T dataset (NRC, younger, physically healthier psychiatric patients possibly more resembling outpatient characteristics). Beyond algorithm validation, the study also quantified the clinical impact of implementing this algorithm as a second-reader screening for incidental aneurysms in the evaluation of clinically acquired cMRI scans. The results highlight a significant risk of incidental aneurysm non-reporting (94.4% for radiologists, 86.4% for neuroradiologists) and demonstrate that the highly sensitive AI algorithm might be beneficial as a second-reader tool. However, the algorithm suffers from extensively high false positive rates (ranging from 61.9% up to 92.5% within the synthesized cohort), and our analysis suggests that a resulting oversensitive detection of incidentalomas in routine cMRI scans would lead to a considerable increase in follow-/work-up imaging workload (cMRI/DSA for 2.17–2.95% and 1.99–2.04% of patients, respectively, summed up to 4–5% when considering only the first follow-up), while additionally identifying only a small proportion of patients with therapeutically relevant aneurysms (0.45–0.74%)—this obviously limits the clinical impact and utility of the current algorithm version. To the best of our knowledge, except for the previous and smaller pilot study [18], this is the first and largest study designed to quantitatively balance this pain–gain ratio of an AI-driven cMRI routine screening, utilizing a large, clinically representative cohort, incorporating a state-of-the-art algorithm, and including a prospective study part with patient consultations and follow-up/work-up evaluations.

Suitable performance comparisons with other AI algorithms in this domain are limited. Lehnen et al. externally validated a previous, less sensitive version of the same vendor’s algorithm using an artificially pathology-enriched cohort, reporting an overall sensitivity of 72.6% and specificity of 87.2% [20]. Other AI solutions for aneurysm detection based on TOF-MRA imaging have also been validated, with sensitivities ranging from 70% to 100%, nevertheless again conducted on pathology-enriched cohorts, which precludes the calculation of prevalence-dependent metrics [21,22,23,24,25]. Regarding the screening background of the study results presented here, the context of the literature needs to be considered, which notes that general aneurysm screening has not yet been established and is currently reserved for a very small group of high-risk patients [31,32]. However, one underlying assumption of this situation is that a screening program would require dedicated medical image acquisition, typically MRI scans, which likely results in unfavorable cost-effectiveness analyses (except for small high-risk subgroups) [17,33,34]. In contrast, the approach of the present study assumes that, with increasingly powerful AI-based image analysis, effectiveness analyses may differ if, rather than acquiring dedicated cMRIs for screening purposes, only those cMRIs already performed for other clinical reasons are algorithmically analyzed in addition to the reporting (neuro-) radiologists’ assessments. The consideration of such an algorithmic implementation, however, leads to classic diagnostic pitfalls, such as primary algorithmic oversensitivity, which may then be further reinforced by medical experts’ confirmation bias, resulting in at least a high diagnostic follow-up burden, but also substantial patient concern, and in the worst case potentially unnecessary invasive procedures. Those phenomena have, for instance, already been observed in a different medical context with the use of conventional CAD systems in mammography screening [35,36,37].

The study results presented here for incidental aneurysm screening similarly suggest that while a small number of clinically relevant aneurysms may be additionally detected, this comes at the cost of disproportionate follow-up efforts (including potentially invasive procedures) and also small or inconclusive findings. Despite its ability to reduce missed aneurysms, the presented AI screening tool therefore imposes a significant strain on clinical resources, and the psychological effects also need to be considered; the long-term follow-up of inconclusive findings might lead to substantial patient concern. To address this currently critical pain–gain ratio in future studies, an algorithmic adjustment to the screening context should be pursued, both generally but also related to individual patient characteristics and subgroups. Differing AI performance results between the 1.5T GRC patients (in the present study presumably also influenced by older, sicker patients and more motion artifacts) and 3T NRC patients suggest substantially increased FPRs with reduced image quality, indicating that automatic algorithmic image analysis should potentially be restricted in such cases. Additionally, an adjustable AI thresholding based on patient-specific criteria should be considered: the highly sensitive detection of very small findings should be possibly reserved for younger and/or high-risk patients (e.g., history of smoking, arterial hypertension, or family history). Implementing adjustable algorithmic thresholding tailored to patient-specific factors could lead to a more favorable evaluation of an AI-based aneurysm screening of routinely acquired cMRI scans.

The limitations of our study include the still relatively small sample size of the non-pathology-enriched cohort in relation to the low disease prevalence and its single-center design, which may limit generalizability, introduce cohort composition bias, and cause the recruitment of reference readers who underwent similar neuroradiology and neurointerventional training. Additionally, a small subset of the 10 GRC reference reading datasets originated from patients under 18 years of age, who may be underrepresented in the algorithm’s training data. Additionally, the reference reading approach, which relied on a subset of data distributed among three neurointerventional experts without cross-validation, did not allow for inter-reader variability analysis. Different field strengths of the NRC (3.0T) and GRC (1.5T) for clinic-specific reasons may have introduced bias but also allowed us to visualize the effects of the field strength and image quality on algorithm performance. The predominantly retrospective nature of the study limits its reflection of real-world prospective AI integration; however, our study aimed to approximate a prospective setting by initially reviewing a retrospectively identified cMRI dataset while simultaneously aiming us to follow up with these patients during the prospective phase of the presented study. Nevertheless, this intended approach was ultimately constrained by low patient follow-up compliance (only 19 out of 82 patients [23.2%] from the overall synthesized cohort and even lower in the GRC), thereby limiting data robustness.

In conclusion, the study results presented here may provide a first data foundation for further research, not only with a technical and clinical focus on adapting and patient-specifically tailoring algorithm performances to potential second-reader screening scenarios but also with an emphasis on subsequent cost-effectiveness analyses.

## Data Availability

Anonymized data-sharing possibilities can be evaluated upon request in coordination with our institutional data protection office.

## Abbreviations

The following abbreviations are used in this manuscript:

ACA	anterior cerebral artery
AchA	anterior choroidal artery
AcomA	anterior communicating artery
AI	artificial intelligence
AI+	MRI with AI detections
AI-	cMRIs without AI detections
AVM	arteriovenous malformation
cMRI	cranial magnetic resonance imaging,
CTA	computed tomography angiography
DSA	digital subtraction angiography
FU	follow-up
GRC	general radiologists’ cohort
ICA	internal carotid artery
MCA	middle cerebral artery
MRA	magnetic resonance angiography
NNS	number needed to screen
NRC	neuroradiologist’s cohort
PCA	posterior cerebral artery
PcomA	posterior communicating artery
PICA	posterior inferior cerebellar artery
PPV	positive predictive value
RFS	reference standard
SAH	subarachnoid hemorrhage
SCA	superior cerebellar artery
TOF-MRA	time of flight magnetic resonance angiography
UIAT score	unruptured intracranial aneurysm treatment score
yo	years old
